# Design of a 3D printed, motorized, uniaxial cell stretcher for microscopic and biochemical analysis of mechanotransduction

**DOI:** 10.1242/bio.057778

**Published:** 2021-02-10

**Authors:** Noor A. Al-Maslamani, Abdulghani A. Khilan, Henning F. Horn

**Affiliations:** Biological and Biomedical Sciences Division, College of Health and Life Sciences, Hamad Bin Khalifa University, P.O. Box 34110, Doha, Qatar

**Keywords:** Uniaxial stretcher, Mechanobiology, 3D printing, Cell stretching

## Abstract

Cells respond to mechanical cues from their environment through a process of mechanosensing and mechanotransduction. Cell stretching devices are important tools to study the molecular pathways responsible for cellular responses to mechanobiological processes. We describe the development and testing of a uniaxial cell stretcher that has applications for microscopic as well as biochemical analyses. By combining simple fabrication techniques with adjustable control parameters, the stretcher is designed to fit a variety of experimental needs. The stretcher can be used for static and cyclic stretching. As a proof of principle, we visualize stretch induced deformation of cell nuclei via incremental static stretch, and changes in IEX1 expression via cyclic stretching. This stretcher is easily modified to meet experimental needs, inexpensive to build, and should be readily accessible for most laboratories with access to 3D printing.

## Abstract (precis)

**Summary:** Description and testing of a 3D printed uniaxial stretcher designed for the microscopic and biochemical analysis of mammalian cells subjected to mechanical stretch.

## INTRODUCTION

Cell biology has historically focused on chemical signaling as the way in which cells interact with their environment. We now appreciate that the mechanical environment also has important consequences for development and disease. Cells respond directly to their physical environment through a number of cellular changes that effect cell and tissue architecture and play a major role in determining cellular fate and function. For example, the mechanical environment can drive the development of pluripotent cells into specific lineages (Engler et al., 2006). Soft environments favor development into neurons (Georges et al., 2006; Ulrich et al., 2009), intermediate stiffness favors differentiation into muscle (Engler et al., 2004; Gilbert et al., 2010), and stiff environments favor differentiation into bone (Engler et al., 2006; García and Reyes, 2005). The nuclear envelope protein Lamin A plays an important role in promoting this mechanically driven differentiation (Swift et al., 2013).

Changes in tissue stiffness, either through changes in the extracellular matrix, or through intracellular alterations are important contributors to aging (Park et al., 2020). Indeed, mechanobiology plays a key role in diseases that are often associated with aging. This includes cancer (e.g. Fischer et al., 2020; Lee et al., 2019), cardiovascular disease (e.g. Atkins et al., 2016; Takahashi et al., 2013) and hypertension (e.g. Bloodworth et al., 2015; Dieffenbach et al., 2018). In addition, mechanics of the nuclear envelope play a key role in a group of diseases collectively known as laminopathies. These diseases include muscular dystrophies, lipodystrophies, premature aging and cardiomyopathies (Burke et al., 2001; Goebel and Warlo, 2000; Stiekema et al., 2020; Worman, 2012). Therefore, there is a real need to understand the effect of the mechanical environment on cell behavior. While significant progress has been made, many questions remain to be answered.

At the core of the mechanobiology related diseases is the nuclear envelope, which is key to mechanotransduction, the process that allows a cell to respond to mechanical stimuli (Graham and Burridge, 2016; Hamill and Martinac, 2001). Mechanotransduction, at least in part, depends on a physical link between the cytoskeleton and the nucleus, which is provided by the LINC (linker of nucleoskeleton and cytoskeleton) complex (Crisp et al., 2006; Gundersen and Worman, 2013). The LINC complex is an essential protein complex for relaying forces to nucleoskeletal proteins such as Lamin A. Lamin A has over 600 mutations that lead to at least 15 distinct laminopathies (de Leeuw et al., 2018). How one protein can play a role in so many different diseases is unknown, but the effect is generally though to occur through three potential mechanisms: structural, gene regulation, and stem cell lineage determination (recently reviewed in Stiekema et al., 2020). The ‘structural hypothesis’ says that lamin mutations alter the structural integrity of the nuclear envelope (NE), thus making the NE less resilient to mechanical stress. This results in increase nuclear damage and cell death when cells undergo mechanical stress (Davidson and Lammerding, 2014; Earle et al., 2020; Lammerding et al., 2004, 2004; Schreiber and Kennedy, 2013; Simon and Wilson, 2013; Stiekema et al., 2020).

A number of approaches have been used to study mechanobiology and mechanotransduction. These include atomic force microscopy, optical tweezers, micropipette aspiration and mechanical stretching (reviewed in Kamble et al., 2016). Cell stretchers is the most heterogeneous category of these tools: some of these stretchers are commercially available such as STREX (Matsugaki et al., 2013) and FlexCell (Callaghan and Williams, 2000; Richard et al., 2007), but the majority of stretchers are custom made. These stretchers can be classified by whether they are designed for static or cyclic stretching, microscopy or biochemical analysis, and uniaxial or biaxial stretching. Several 3D printed models of stretchers have been recently described that are ideally suited for static stretching. These stretchers are inexpensive to manufacture and can be used for microscopy and biochemical analysis. Stretching is performed by manually adjusting a cog or screw that changes the strain on the membrane (Daulagala et al., 2020; Mayer et al., 2019), or by adding a weight to extend the membrane (Isermann et al., 2012). These stretchers can be used for biaxial (Isermann et al., 2012; Mayer et al., 2019) or uniaxial stretching (Daulagala et al., 2020; Mayer et al., 2019).

For cyclic stretching, especially over extended periods of time, motorization is required. Several stretchers have been described recently that make use of vacuum (Huang and Nam-Trung, 2013) or motors (Boulter et al., 2020; Huang et al., 2010; Shiwarski et al., 2020) to drive the stretching device. Some of these stretchers are ideal for high-resolution live cell imaging (Huang and Nam-Trung, 2013; Huang et al., 2010; Shiwarski et al., 2020), while others excel at biochemical analysis and fixed cell microscopy (Boulter et al., 2020). One of the key differences between these capabilities is the number of cells a stretcher can accommodate, with larger number of cells being more amenable to biochemical analysis.

Use of these various stretchers has shown that cells respond to mechanical stress. For example, cyclic mechanical stretching leads to cellular reorientation and a subsequent nuclear rotation and elongation (Anno et al., 2012; Brosig et al., 2010; Hoffman et al., 2020). Mechanosensitive transcription factors such as YAP/TAZ and MRTF-A translocate from the cytoplasm to the nucleus in response to mechanical stretching which results in transcriptional activation of gene expression such as the immediate early response gene IEX1 (Hoffman et al., 2020; De Keulenaer et al., 2002; Ohki et al., 2002; Schulze et al., 2003). The LINC complex proteins are key mediators for the different mechanisms of mechanotransduction, and YAP nuclear translocation has been shown to be dependent on the LINC complex protein Nesprin1 (Driscoll et al., 2015).

Our interest in mechanobiology led us to require a stretching device that was able to do live-cell imaging and biochemical analysis. That is, the cell culture area needed to be large enough for growing sufficient cells for biochemical analysis, but also have optical properties that allowed for microscopic analysis. We needed a device that was able to perform cyclic stretching and was simple and inexpensive. Finally, we wanted to exert relatively even, uniaxial force across the entire field of stretch, and allow for easy adaptation to different experimental needs. None of the stretchers above met all of our requirements, which led us to employ 3D-printing to create a stretcher compatible with live-cell imaging and biochemical analysis with control capabilities for uniaxial static and cyclic stretching. As with many of the previously published stretching devices, we used elastomeric polydimethylsiloxane (PDMS) as membrane material to culture and stretch the cells. Using this stretcher, we were able to confirm stretch-induced nuclear deformation by live-cell microscopy, and we were also able to show an increase in IEX1 expression by quantitative real-time PCR in cells subjected to cyclic stretching.

## RESULTS

### Stretcher frame

**Fig. 1.**
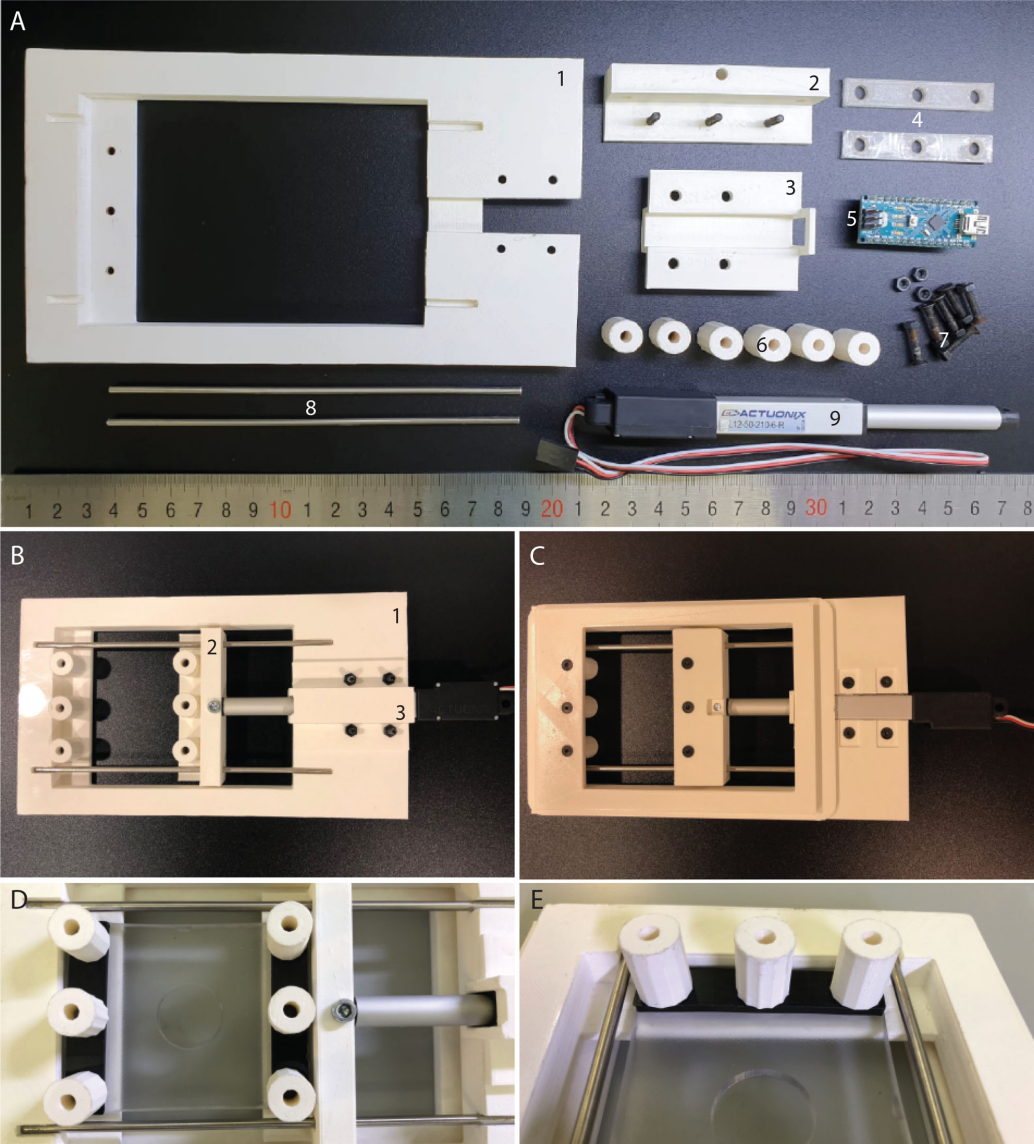
**Stretcher components and assembly.** (A) All plastic components for the stretcher were 3D printed in PLA. The stretcher frame (1) is designed to seat into the stage of a Nikon A1R confocal microscope. It was also designed to accept a motor (9) and motor cover (3), two metal rods (8) and has holes for a number of bolts (7) to attach the motor as well as the PDMS membrane. The ‘motor hand’ (2), accepts the motor arm into a pocket, and is then attached via a screw. The motor is controlled using the Arduino Nano (5). The PDMS membrane is attached to the stretcher using three bolts on each side, one through the motor hand (2, with bolts shown), and the other in the stretcher frame at the end opposite from the motor (shown in A with no bolts). The membrane is secured by placing washers (4) over the PDMS and bolts, and then securing everything with nuts that have been set into 3D printed cylinders (6). (B) A top-down view of the assembled stretcher without PDMS membrane. Note the metal rods have been pushed into the frame (1) and the motor hand (2) is guided along the metal rods. The motor is mounted to the frame with help of a motor cover (3). (C) View of the assembled stretcher from below. (D) Assembled stretcher with PDMS membrane mounted. (E) Close-up of the membrane attachment site. Note the washer on top of the PDMS helps not only to secure the membrane in place, but also distribute the pressure from the screws evenly across the PDMS.

The frame of the stretcher was designed to fit into the stage of the Nikon A1R confocal microscope. [The design has also been successfully modified to fit the stage of a Zeiss LSM780 (data not shown), and in principle, can be adapted to fit most inverted microscopes.)] Using the dimensions of the slide holder stage insert for the Nikon A1R, the stretcher frame was modeled in 3D using Autodesk^®^ Inventor^®^ software. A space for seating a linear actuator to apply mechanical force to a flexible membrane was included (Fig. 1A). Also included were spaces for two 3 mm stainless steel rods placed parallel to each other to support and stabilize the motor hand and prevent horizontal and vertical twisting of the PDMS membrane (see Fig. 1A and B, component 2). To allow for coupling of the PDMS membrane and the motor to the stretcher frame, 3 mm holes were included in design. These holes were made to fit M3 flat head hex socket bolts (16 mm). The assembled stretcher is shown in Fig. 1B from the top view and Fig. 1C from the below view. In order to secure the PDMS membrane to the frame and the motor hand, rectangular washers, 55 mm in length and 3 mm in depth were printed to be placed on top of the PDMS to distribute the force of the screws (Fig. 1A, component 4). The PDMS and motor assembly were all fixed using 3 mm flat head bolts and screws. For ease of mounting and dismounting the membrane, nut fasteners were also printed to allow for the tool-less tightening and securing of the membrane to the stretcher. The fasteners were designed to allow the snug seating of a M3 nut within (Fig. 1A, component 6 and Fig. 1D and E). The assembly of the PDMS membrane with the stretcher is shown in Fig. 1D and E. All plastic components were printed using the Ultimaker 3 Extended 3D printer with polylactic acid (PLA) as material. PLA is a bioplastic, derived from corn, is biodegradable, does not emit toxic fumes when printed, and has excellent mechanical rigidity (Pakkanen et al., 2017). All STL (stereolithography) files for the stretcher are uploaded to a free open source sharing platforms (https://github.com/Henning-Horn).

### Actuator and controller

**Fig. 2.**
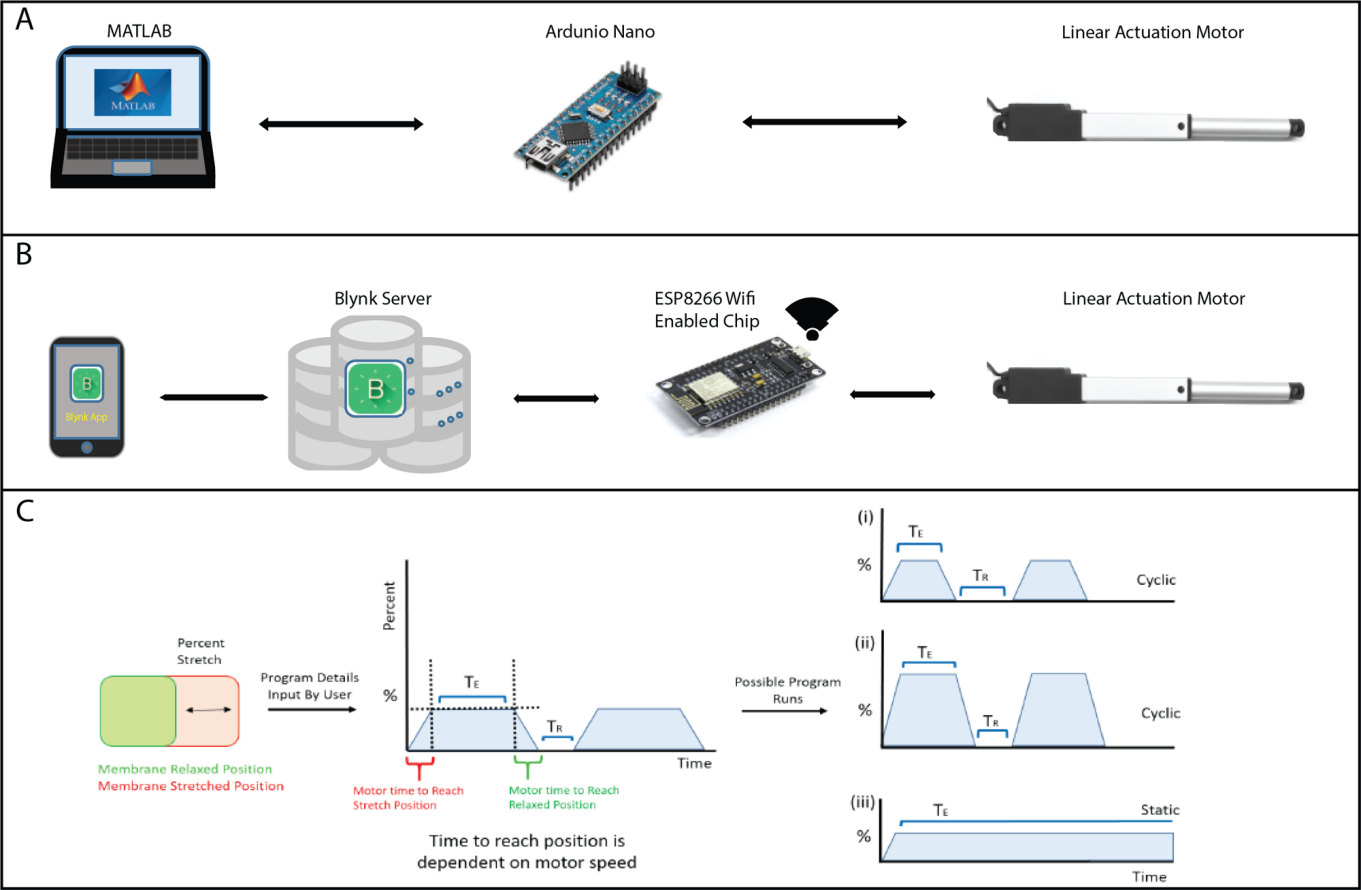
**Stretcher control and actuation specifications.** (A) A linear actuation motor can be controlled either from a laptop via an external Arduino Nano board or (B) via wifi from any android or IOS device through Blynk IoT platform. (C) The percent stretch is the difference (in mm) between the stretched and relaxed membrane dimensions in the x-axis, divided by the relaxed membrane dimensions. The user inputs the following variables: percent stretch, time to hold stretch (T_E_), and rest period between stretches (T_R_). Times to transition between unstretched and stretched is dependent on motor specifications. For illustration, three possible stretching scenarios are shown on the right: (i) a cyclic stretch with low percent membrane stretch, (ii) a cyclic stretch with high percent membrane stretch and shorter rest intervals (T_R_) between stretching, and (iii) a static stretch.

A micro linear actuator (Actuonix L12-50-210-6-R) was incorporated into the design to create the mechanical forces needed to stretch the PDMS membrane (Fig. 1A, component 9). This motor has a stroke length of 50 mm with slow speed, but high force, operates on 6 volts and has a digital servo interface, in which the value of 1 ms pulse command retracts the motor hand and a 2 ms pulse signal extends it. The motor is capable of withstanding temperatures as high as 50°C and has an ingress protection of 54, which indicates that the motor is protected against dust and splashes of water from any direction. Thus, it is suitable for operation inside a cell culture incubator. The motor is controlled from a laptop via an external microprocessor, an Arduino Nano (Fig. 1A, component 5). This controller is 18×45 mm in size and is powered via a Mini-B USB cable. The motor is powered through DC pin no. 27 for +5 V, grounded at pin no. 29, and motor communication is done through digital pin no. 9. The Arduino Nano is both programmed and controlled through a program on MATLAB R2016b (The full code can be found https://github.com/Henning-Horn), in which the value of 0 retracts the motor hand and a value of 1 extends it. The program allows for user interface, requesting details of stretch time, cyclic or static, pause in cyclic stretch and the percentage of arm movement. Upon receiving this information from the user, the motor arm moves accordingly, which allows for complete manipulation and control of stretching procedure by the user (Fig. 2A).

### Wireless control

We also designed a slightly more user-friendly controller interface using the Blynk IoT app. This is an optional modification that requires the use of a wifi enabled controller chip, and access to Blynk servers (Fig. 2B). The Blynk IoT platform allows the building of an interface that controls the stretcher wirelessly through an application on android and IOS devices (Fig. S1A). Using an open secured cloud network and an authentication token, the Blynk platform can send and receive messages to and from the stretcher. To use BLYNK IoT, a Blynk compatible hardware module that allow for wifi configuration and communication had to be used. The Arduino Nano was replaced with the ESP8266 wifi. The ESP8266 wifi controls the stretcher motor and was programmed to allow for communication between Blynk application and the stretcher. The stretcher program was written and uploaded to the ESP8266 through the Arduino IDE. The detailed code can be found at https://github.com/Henning-Horn. Using this platform, we were able to perform both cyclic and static stretch as well as control the initial position of the motor, percent movement, and observe the status operation of the stretcher, all wirelessly through any android/IOS device (Fig. S1B and C). While this control modality is more user friendly, we found that we gave up control of some stretching parameters. In particular, in cyclic stretch mode, the hold-of-stretch duration and the duration of rest between stretches can be varied in the MATLAB code. However, with the Blynk App, we found that varying these values caused the app to disconnect from the server and the program to stop running. As a result, we programmed a fixed loop-time-function instead of delays between stretch and relaxation. Users should decide if a wired or wireless approach is more appropriate for their experimental needs.

### Variable stretching protocols

Fig. 2C indicates the variable stretch parameters that can be performed by this stretcher. The main variables are time of stretch (T_E_), time of rest (T_R_) and the amplitude or percent stretch. Using these three variables, the stretching protocol can be adjusted to fit most experimental needs. The limiting parameters are that the percent stretch cannot exceed the maximum stretch of the PDMS membrane, and that the speed of the actuator motor is fixed. The Actuonix actuator model we used has a gear ratio of 210:1, which has a no-load maximum speed of 6.5 mm/s. Fig. 2C also shows an example of cyclic and static stretch protocols that are achievable with this stretcher. Movie 1 shows the stretcher performing a cyclic stretch.

### PDMS Membranes

Several types of flexible support materials have been used in biomechanical stretchers (Huang and Nam-Trung, 2013; Lee et al., 2004; Mawatari et al., 2010; Nagai et al., 2012; Trappmann et al., 2012; Vanderploeg et al., 2008). We chose to use PDMS, a flexible substrate with good biocompatible properties that has been used successfully by other groups for similar applications (Friedrich et al., 2017; Huang and Nam-Trung, 2013; Lee et al., 2004). The design of the culture well within the PDMS structure was driven by the need to have a multi-application stretcher: to be able to grow enough cells for biochemical analysis and also have enough optical clarity of the culture surface to allow microscopic investigation. To minimize optical interference of the PDMS, the targeted membrane thickness of the cell culture surface was 150–200 µm. This thickness impacted the upper limit of the culture well area since the larger the area, the greater the weight of the culture media, and the greater the membrane distension due to the weight of the media. We examined a number of different sizes for the desired ∼170 µm membrane thickness and chose to proceed with a circular membrane with a 20 mm diameter (area of 314.16 mm^2^) and a square membrane with a 20×20 mm size (area 400 mm^2^), as we found the convexing of the membrane to be acceptable with these sizes.

**Fig. 3.**
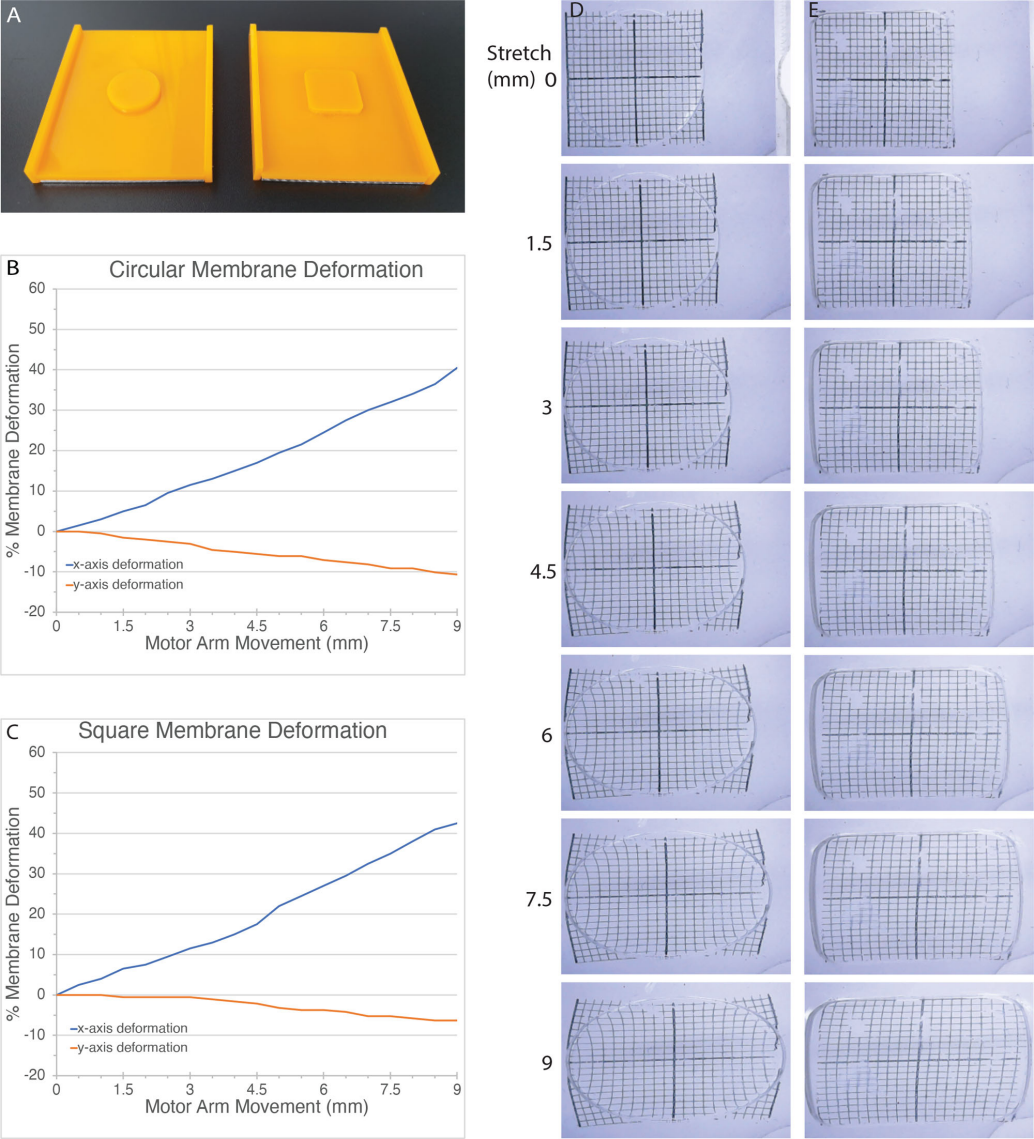
**PDMS membrane characterization.** (A) Plexiglass molds for PDMS casting showing a circular well (left) and a square well (right) casting mold. (B) Analysis of circular PDMS membrane deformation in x and y in response to stretch. Percent x/y deformation is shown on the y axis, motor arm movement is shown on the x-axis. (C) Analysis of square PDMS membrane deformation in x and y in response to stretch. Percent x/y deformation is shown on the y-axis, motor arm movement is shown on the x-axis. (D) Intramembrane deformation of circular PDMS membrane is shown for 0 to 9 mm stretch. A 20×20 1-mm grid was laser printed on acetate and transferred to the PDMS using brief application of heat. (E) Intramembrane deformation of square PDMS membrane treated in the same way as the circular membrane in D.

Plexiglas molds were used to cast the PDMS. The dimensions of the mold were such as to create a cast PDMS with dimensions of 71×59 mm, with either a circular or square culture area (Fig. 3A). To achieve the desired thickness of the well bottom, the edges of the casting tray were 3.3 mm tall, and the well pattern was 2.9 mm tall, both as measured from the surface of the casting tray. This differential of 0.4 mm resulted in membrane thickness within our target range (0.15–0.2 mm). When pouring the PDMS into the molds, some PDMS tended to spill over the edges of the mold before curing, leaving a less than 0.4 mm layer of PDMS on top of the circle or square. The molds were designed to have the same length as a small agarose gel from Bio-Rad, so that we could use the agarose gel casting system from Bio-Rad to seal the edges of the molds (Fig. S2). The benefit of this approach over a mold that is permanently sealed on all four edges is that it is easier to release the membrane from the mold once the PDMS has cured. The PDMS (Sylgard^®^ 184) was mixed in a 10:1 ratio of base to curing agent. This ratio has been shown to provide an enhanced cell adherence, growth and viability (Lee et al., 2004). Since temperature directly affects the curing rate of PDMS, all membranes were cured at room temperature for 48 h, which had the benefit of allowing trapped bubbles in the PDMS to be released. It has been shown that curing PDMS at room temperature (∼24°C) for 48 h yields PDMS with a Young's modulus of 1.32±0.07 N/mm2, providing the desired elastic properties (Johnston et al., 2014). The support PDMS had a 3.30 mm thickness, while the layer at the bottom of the well ranged in thickness from 150–220 µm. We found this to be adequate for visualization on an inverted microscope. Mounting holes were punched into the PDMS to line up with the 3 mm bolts on the stretcher. This allowed the membrane to slide onto the screws extruding from the stretching device (Fig. 1).

### Membrane characterization

To understand how the stretching device would impact cells, we characterized the stretching characteristics of the PDMS membranes with round and square culture areas. Both membranes consisted of a 10:1 ratio of base to curing agent and were cured at room temperature for 48 h. Two types of characterizations were performed: (1) a measure of the change in X and Y axis displacement as the membrane was stretched; and (2) a visual inspection of the intra-well deformations as the membrane was stretched. Our objective was to have a membrane with good linear stretch characteristics, and a relatively even distribution of the stretch throughout the entire well.

The circular well was characterized first. The stretch was initiated by adjusting the motor elongation so the well was unstretched with a diameter of 20 mm. The membrane was then stretched in 0.5 mm increments. With each incremental step the well deformation was measured and plotted (Fig. 3B; Fig. S3). When mapping motor arm movement to the PDMS well deformation, there was a relatively linear relationship between the motor arm movement and x-axis deformation. And generally, there was a smaller inverse linear relationship between motor arm movement and y-axis deformation. (Fig. 3B). Both x- and y-axis deformation showed changes in slope that appeared to correlate to each other. When there was a greater change in y-axis deformation, this correlated to a lower deformation rate in the x-axis. As a result, while the overall relationship between the motor arm movement and x-axis deformation of the well was linear, we noted that the slope of the x-axis deformation was not constant. Measurements are shown up to 9 mm motor arm retraction.

A similar analysis was performed for the square well. The initial design of the square membrane had sharp corners (not shown), but we found that these sharp corners were the initiation sites of catastrophic failure (tearing) for the membrane. By rounding the corners, the design was more resilient to stretching. The initial x- and y-axis measurements for the square membrane were 20 and 18.9 mm, respectively. The membrane was stretched in 0.5 mm increments, each increment was measured and plotted for changes in x and y. Similar to the round well, the square well also had a relatively linear relationship between x- and y-axis deformations compared to motor arm movement (Fig. 3C; Fig. S3). However, a key difference is that the y-axis deformation was always less at comparable levels of stretch. For example, at 3 mm stretch, the square membrane had 0.5% y-axis deformation compared to 3% for the round membrane. At 4.5 mm stretch, the square membrane had a 2.1% deformation in the y-axis, compared to a 5.6% in the circular membrane. Thus, the square membrane has a better x-axis deformation capability, with less of a y-axis deformation when compared to the round membrane.

### Intra-well deformation

We performed a basic visual analysis of intra-well deformation by printing a 20×20 1-mm grid onto a piece of acetate, which was then transferred onto the PDMS by brief application of heat. This allowed for ready visualization of the different areas of the well in response to stretch. At 3 mm stretch, the circular well showed uneven distribution of the stretch within the well, with the peripheral edges along the x-axis showing a greater degree of distortion compared to the center of the well, which appeared almost unstretched. As the degree of stretching increased, this disproportionate distribution became increasingly apparent (Fig. 3D). By contrast, the square well appeared to have a more even distribution of the stretch throughout the membrane (Fig. 3E). Uneven stretch was apparent at the x-axis periphery compared to the center even in the square membrane, but this was substantially less pronounced than in the circular membrane.

The uneven stretch distribution of the circular well raises the obvious concern that any population of cells cultured on these circular membranes will have distinct sub-populations that are experiencing very different mechanical stretch. Cells growing along the periphery of the circle will receive greater stretch than cells growing in the center of the well. As a result of this intra-well distortion analysis and the amount of y-axis deformation seen in square versus round membranes, we continued our analysis of the stretcher using square membranes.

### Cell culture on PDMS membrane

To prepare the membranes for cell culture they were washed with deionized water and sterilized with 70% ethanol. Membranes were then coated with 2 µg/ml fibronectin in phosphate buffered saline (PBS, pH 7.5), and incubated at 4°C overnight within parafilm sealed petri dish to prevent the solution from evaporating. After 24 h, membranes were washed once with PBS and once with DMEM. 3×10^5^ U2OS cells were seeded directly onto the PDMS membrane and incubated overnight at 37°C and 5% CO_2_. When not mounted to the stretcher, the thin membranes sagged under the weight of the media and caused uneven seeding of cells. Therefore, supports were 3D printed which allowed not only for easier handling of the PDMS but also kept the bottom of the membranes from sagging. In addition, it kept the membranes from adhering to the surface of the tissue culture dish that we used to house the membrane while in culture (Fig. S4).

### Flexibility and multi-purpose functionality of the stretcher

One of the main aims for designing this stretcher was to develop a tool that allowed for investigation of the LINC complex in defining the nuclear responses to mechanical strain. Consequently, a necessary feature was to be able to use the stretcher in microscopy as well as biochemical analyses with the ability to perform both cyclic and static stretching. The relatively small footprint of the stretcher allowed for easy operations inside a standard CO_2_ incubator for longer periods of cyclic stretching, and also fit onto a confocal microscope stage. Therefore, the stretcher can be used for both biochemical and structural morphology studies. The thin bottom wells facilitate microscopy of live cells during stretching or immunofluorescence staining of fixed cells. The size of the well is also large enough to allow cells to be subjected to biochemical analysis. From U2OS cells, we routinely obtain around 5 µg of total RNA per membrane.

### Increase in nuclear area in response to stretching

**Fig. 4.**
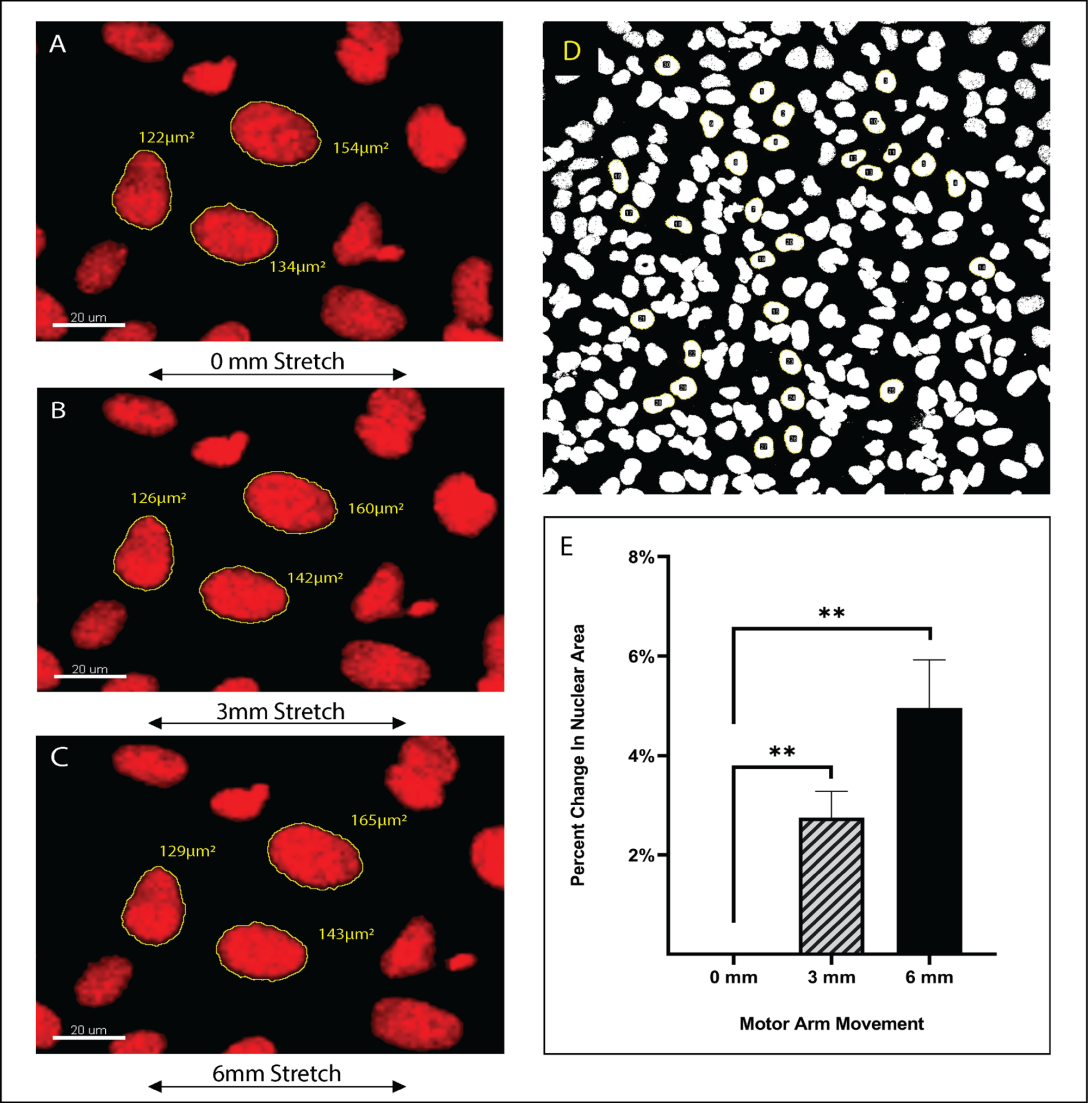
**Microscopic analysis of stretched cells.** Representative images of three nuclear area measurements from the same membrane at (A) relaxed state, (B) 3 mm stretch, and (C) 6 mm stretch. (D) FIJI-ImageJ analysis of nuclear area. Hoechst stained nuclei were threshold adjusted and the area was measured using the ROI manager. Statistical analysis of change in nuclear area in response to stretching, from unstretched (0 mm) to 3 mm (*P*-value =0.0066) and from unstretched (0 mm) to 6 mm *P*-value =0.0067 (E). A total of 276 nuclei were analyzed in three different membranes and statistical analysis was performed by unpaired *t*-test.

Previous studies have shown that mechanical stretching leads to changes in nuclear shape and size (Anno et al., 2012; Brosig et al., 2010; Hoffman et al., 2020; Mayer et al., 2019). To verify that our stretcher could be used to study the impact of stretching on cells, we subjected U2OS cells to incremental static stretch and measured changes in nuclear area. U2OS cells were seeded onto fibronectin coated PDMS membranes and incubated for 24 h at 37°C and 5% CO_2_. After 24 h, NucBlue™ (Hoechst 33342) live cell stain was added to the cells, the membranes were mounted onto the stretcher and the stretcher was fitted to the confocal microscope stage. Images were collected at 0, 3 mm and 6 mm motor arm movement, corresponding to 0%, 11.5% and 24.5% deformation in the x-axis of the membrane, respectively. At each position the same field of cells was imaged. The nuclear area of some 270 cells in three different membranes was measured using FIJI/ImageJ image software. Fig. 4A, B and C are a representative image of measurements recorded. We found that when nuclei were immediately adjacent to each other, FIJI often recognized the two nuclei as one, giving an inaccurate area measurement. As a result, nuclei were randomly selected in a field of view for analysis, but those that were immediately adjacent to other nuclei were excluded (Fig. 4D). The average change in nuclear area was graphed and showed that mechanical stretching induced a significant increase in nuclear area (Fig. 4E). Stretching the membranes to 11.5% x-axis deformation resulted in an average increase in nuclear area of 2.75% compared to the relaxed state and the 24.5% x-axis deformation induced a 4.96% increase in nuclear area compared to the relaxed state.

### Induction of *IEX1* expression

Expression of *IEX1* has been shown to be induced in several cell types in response to mechanical stress (De Keulenaer et al., 2002; Ohki et al., 2002; Schulze et al., 2003). Therefore, we sought to use IEX1 as a marker to confirm that our stretcher is able to affect the transcriptional program of cells. Equal numbers of U2OS cells were cultured on two fibronectin-coated PDMS membranes. The membrane thickness of control (unstretched) and experimental (stretched) samples were within 20 µm of each other (within 5 µm of each other for three of the four experimental runs: control 1: 155 µm, stretched 1: 152 µm; control 2: 155 µm, stretched 2: 152 µm; control 3: 200 µm, stretched 3: 219 µm; control 4: 156 µm, stretched 4-1: 152 µm, stretched 4-2: 155 µm). Twenty-four hours after seeding, one population of cells was subjected to a cyclic stretch for three hours at 17% strain (4.5 mm motor arm movement) and 0.5 Hz frequency rate. That is, a complete cycle of the membrane going from unstretched to stretched to unstretched lasted 2 s. This stretch regime (17% strain at 0.5 Hz) is in line with previously published stretching studies (Bianchi et al., 2019; Chancellor et al., 2010; Hoffman et al., 2020; Mayer et al., 2019; Travaglini et al., 2020) and is in the physiologically relevant range. For endothelial cells, previous studies have used a 10% strain with a frequency of 1 to 1.2 Hz (Barron et al., 2007; Livne et al., 2014). For airway smooth muscles, the physiological stretch protocol was 12% strain at 0.5 Hz (Asano et al., 2018).

**Fig. 5.**
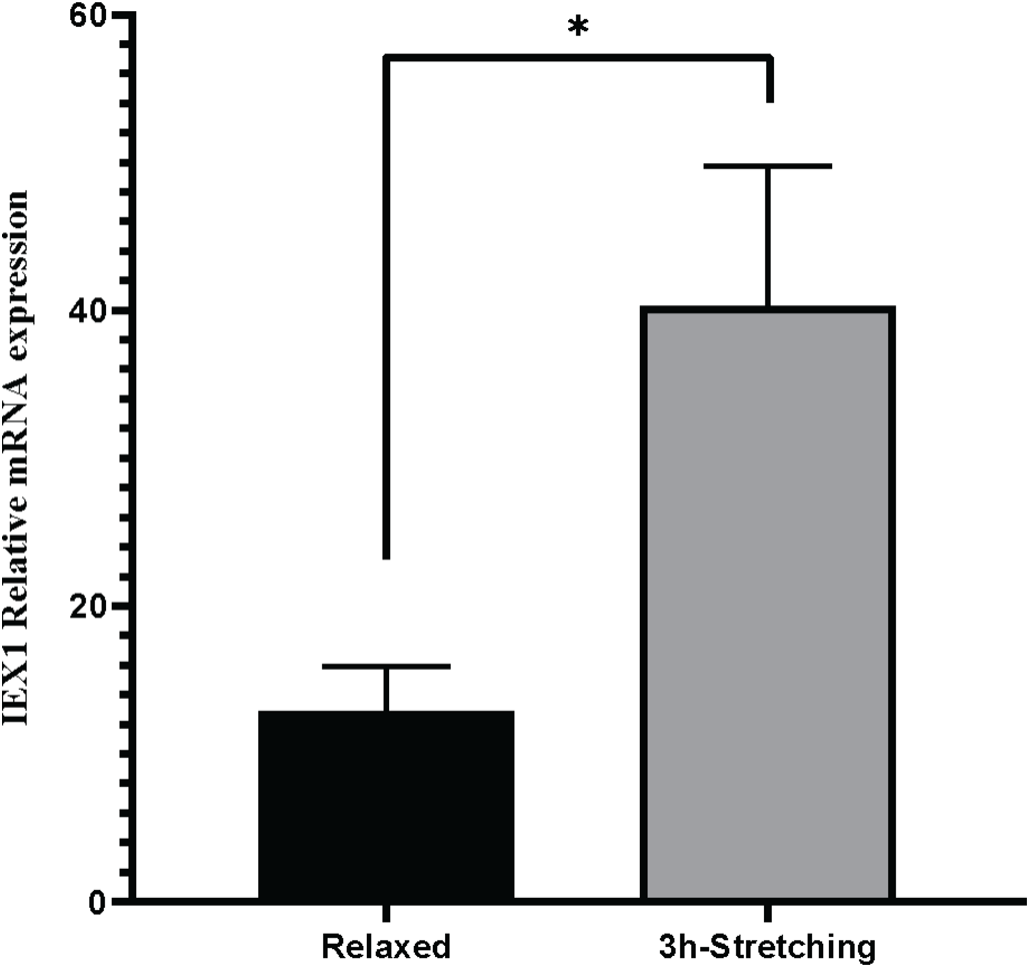
**Real-time PCR analysis of stretched cells.** *IEX1* relative expression levels in U2OS cells at relaxed state (black column) and cells stretched for three hours at 4.5 mm motor arm movement and 0.5 Herz (grey column). Data shown are five biological replicates analyzed by unpaired *t*-test. (*) *P*-value =0.0251.

After 3 h of stretching, cells were harvested for RNA. *IEX1* expression was evaluated by real-time PCR using *PGK1* as a reference for normalization. As expected, cyclic stretching resulted in a statistically significant (*P*=0.025) upregulation of the *IEX1* gene expression (Fig. 5). The gene expression level of *IEX1* was about twofold higher in the cyclic stretched cells compared to the unstretched controls.

## DISCUSSION

We have described the design, building, and testing of a uniaxial stretching device that allows for microscopic and biochemical analysis of mammalian cells in both static and cyclic stretching protocols. Applying mechanical force to cells is a key way to understand the underlying changes that contribute to the mechanobiological aspects of disease pathologies. Uniaxial stretching devices have previously been used to study mechanobiology. Some are commercially available (Flexcell, Strex, Electron Microscope Sciences, etc.; Callaghan and Williams, 2000; Matsugaki, Fujiwara, and Nakano, 2013; Richard et al., 2007), and many labs have designed and built their own custom stretchers (Boulter et al., 2020; Daulagala et al., 2020; Huang and Nam-Trung, 2013; Huang et al., 2010; Isermann et al., 2012; Mayer et al., 2019; Roth et al., 2015; Shiwarski et al., 2020). Our system contributes to this line-up of stretchers in that it has a very simple design, with easy adaptation to experimental needs, and relatively low cost of entry. Assuming access to a 3D printer, the fabrication costs of this stretcher comes to around USD $100, the bulk of which ($70) is the actuator motor (Table S1). This makes it the lowest cost stretcher with cyclic stretching capabilities.

In this study, we report the effects of cyclic stretching for 3 h, where IEX1 expression is increased twofold. We have also performed cyclic stretching for up to 20 h. Consistent with IEX1 being an early response gene (De Keulenaer et al., 2002; Schulze et al., 2003), there is no significant difference in IEX1 expression between unstretched and stretched cells at 20 h (data not shown), indicating the importance of using the correct duration of stretching for the biological question being addressed. Indeed, a recent study investigating changes in heterochromatin in response to cyclic stretching showed that the duration of stretch, as well as the strain levels caused distinct cellular responses, with 5% strain having different cellular effects than 40% strain (Nava et al., 2020). In addition, at 40% strain, some effects of stretch were seen early, but then lost by 6 h of stretching (Nava et al., 2020).

We also show a clear increase in nuclear area of cells that are stretched statically. This is consistent with several published reports, where mechanical stretching leads to nuclear distention when cells are stretched (Chancellor et al., 2010; Hoffman et al., 2020; Mayer et al., 2019; Nagayama and Fukuei, 2020). The transmission of strain from the cell adhesion points to the nucleus is dependent on a functional LINC complex (Alam et al., 2014; Lombardi et al., 2011; Mayer et al., 2019). In addition, the amount of nuclear stretch we observed in our static stretch is in line with what has been recently reported for uniaxial static stretching (Mayer et al., 2019). In our case, as well as previous reports, the amount of nuclear strain is relatively small compared to the amount of mechanical strain placed on the cells. This is consistent with the nucleus being stiffer than the cytoskeleton, and that most of the mechanical strain is absorbed by the cytoskeleton (Isermann et al., 2012; Wang et al., 2018).

We found several parameters to be essential for the consistent performance of this stretcher. The most important variable appears to be the thickness of the membranes. While the casting process was standardized as much as possible, membranes thickness in our hands fluctuated from 150 µm to 220 µm. The difference in this thickness of the culturing surface has potential to affect the cell characteristics, as the thicker membrane offers more resistance to cellular strain than the thinner membrane. Indeed, substrate stiffness plays a key role in differentiation and cellular behavior (Li et al., 2013; Piao et al., 2017; Swift et al., 2013). As such, we have found variation in transcriptional responses when comparing different runs of stretching experiments. To address this challenge, all membrane thicknesses were measured and only membranes that were within 20 µm thickness of each other, (ideally, within 5 µm of each other) were used as experimental/control pairs. This approach yielded more consistent results. We are currently exploring the option of removing the membrane from the well area and attaching a commercially available PDMS sheet to the remaining PDMS frame. This would standardize the thickness of the well, and we predict further reduce some of the inter-experiment variations we currently see.

A second parameter that likely plays a role in the consistent performance of the stretcher is the cells themselves. We found that adopting a strict plating to stretching protocol (i.e. always seeding at the same density at the same time before stretching) was critical for decreasing variation between experimental runs. We anticipate the changes in cell–cell contact and heterogeneity in cell cycle stages can modulate the cellular response to physical stretch.

A potential application of this stretcher that was not tested is its use for differentiation. Several studies have shown that substrate stiffness can direct the differentiation of pluripotent stem cells (Darnell et al., 2018; Engler et al., 2006; Evans et al., 2009). As a future direction, it could be interesting to test whether varying the membrane stiffness through the application of different levels of static stretch could drive the differentiation of stem cells into specific lineages. This could potentially reduce the need for some chemical growth factors, and/or synergize with specific differentiation cocktails to yield a higher purity of the desired cells population.

Our stretcher allows for live-cell imaging under static as well low frequency cyclic conditions. Currently, we mark a field of view and then manually move the microscope stage to find the same field of view under stretched conditions. Because the actuator we used is fairly precise, (±0.2% at 12% strain), we can also use the X-Y coordinates of the microscope stage to locate fields of view under rest and stretch conditions. We realize that this approach is not feasible for live-cell imaging during high-frequency cyclic stretching if the cells are to be imaged in each cycle at rested and stretched positions. To expand the application potential of this stretcher, we are considering implementing an automatic control of the field of view. This can be achieved by controlling the Zeiss confocal microscope stage through the Zeiss-MATLABs [Open Application Development (OAD)]. This would in principle allow cells to be followed and imaged in real time with the stretch.

In conclusion, we describe a uniaxial stretcher for mammalian cells that is suitable for mechanobiology studies. We show that this stretcher can be used for microscopic as well as biochemical analysis of mechanically stretched cells. We believe that the relatively low cost and flexibility in application makes this an attractive tool for laboratories interested in mechanobiology.

## MATERIALS AND METHODS

### Plexiglas casting tray and PDMS membrane curing

A 3 mm thick Plexiglas was cut to two pieces of 7 mm ×71 mm, and one piece of 71×59 mm, the parts were glued to form a 71 mm×59 mm tray, with the 7 mm ×71 mm on the sides (Fig. 3A). In addition, a 3 mm thick 20 mm diameter circle or 20×20 mm square were glued to the center of the tray to form culturing wells. WELD-ON® 4 acrylic glue (SCIGRIP smart adhesive solutions), was used to glue the components together. Sylgard® 184 silicon elastomer kit (Dow Corning) was mixed in a 10:1 ratio of base to curing agent. The two agents were mixed for about 2 min in a 50 ml falcon tube and settled for 5 min before pouring the mixture in the tray. Then, the Plexiglas tray was placed into a gel-casting tray (Bio-Rad) on top of the balance and 11.8 g exactly of the PDMS mixture were poured into the tray to generate membranes with 150–200 µm thickness in the wells. Finally, the PDMS mixture was allowed to cure for 48 h at room temperature.

### PDMS coating and cell culture conditions

U2OS cells were obtained from ATCC^®^ (HTB-96™) and grown in DMEM high Glucose (Gibco), 10% fetal bovine serum (Gibco), 100 units/ml penicillin, and 100 µg/ml streptomycin, and incubated at 37°C and 5% CO_2_. Cells were routinely tested for microplasma contamination. PDMS membranes were washed with d.H_2_O, then 70% ethanol for sterilization. Membranes were coated with 2 µg/ml fibronectin (Human Plasma Fibronectin, 33016-15 Gibco Thermo Fisher Scientific) in final volume of 1 ml phosphate buffered saline (PBS). The PDMS membrane was incubated at 4°C overnight within parafilm-sealed petri dish to avoid drying. The membrane was then washed once with 1 ml of PBS and once with 1 ml complete DMEM medium. 3×10^5^ U2OS cells were seeded onto the PDMS membrane and incubated for 24 h at 37°C and 5% CO_2_.

### 3D printing parameters

Files for 3D printing were generated on Autodesk Inventor software (https://www.autodesk.com/products/inventor/). All printing was performed on an Ultimaker 3 extended 3D printer. The main nozzle used for printing was 0.4 AA. The nozzle for printing support material was 0.4 BB. The support material was water-soluble polyvinyl alcohol (PVA). All models were printed with a brim of 7 mm around the model to establish a filament flow, allow for bed adhesion of the model, and hold down the edges of the model. All components were printed in polylactic acid (PLA). The material printing temperature was set to 205°C, the build plate temperature to 60°C with a 100% flow of material from nozzle. Material diameter is automatically set to 2.85 mm filament diameter. Print speed was set to 70 mm/s, travel speed 250 mm/s, print acceleration to 4000 mm/s2, travel acceleration to 5000 mm/s2, print jerk 25 mm/s travel jerk 30 mm/s. The models where removed once the build plate has completely cooled to room temperature to prevent model bowing due to non-gradual temperature drop. Objects that were printed with support material were kept in water after they were cooled to dissolve the support material. If support material was not completely removed in 4 h the extra material was extracted manually without damaging model. All models were built with layer height of 0.2 mm, wall thickness of 1 mm and top/bottom thickness of 1 mm. PLA and PVA filaments were purchased from Ultimaker.

### Confocal microscopy and nuclear area measurements

Z-stacks were captured for the same field at 0, 3 mm and 6 mm motor arm movement using the Nikon A1R confocal microscope. Images were analyzed with FIJI-ImageJ software (Free download from https://imagej.net/Fiji) using maximum intensity image projection of each z-stack. Images were converted into threshold images to be able to use the ROI manager to measure the area. All images were treated identically to avoid introduction of bias. For Fig. 4A–C, the FIJI maximum intensity projections were saved as TIFF files, imported into Imaris Viewer (Free download from https://imaris.oxinst.com/imaris-viewer) and visualized in the default MPI setting. The nuclei were picked manually then the area was measured and compared for each nucleus at the different stretching positions for the same membrane. A total of three membranes were analyzed with an average of 92 nuclei per membrane. Statistical analysis was performed on GraphPad prism software (https://www.graphpad.com/scientific-software/prism/) and *P*-values were calculated using unpaired *t*-test.

### Real-Time PCR

RNA was extracted using the RNeasy Plus Mini Kit (Qiagen #74136) following the manufacturer's instructions. Briefly, 350 µl of RLT buffer was added directly to the PDMS well after stretching. This was swirled gently for 10 s, and cell lysates were carefully transferred to RNase free Eppendorf tubes for further processing. 500 ng of RNA was used from each sample to prepare cDNA using the High-Capacity cDNA Reverse Transcription Kit (Thermo Fisher Scientific #4368813) following the manufacturer's protocol. The cDNA was diluted 1:5. 5 µl of the diluted cDNA were used for each RT-PCR reaction. The following primers were used:

PGK1-F: GGA GAA CCT CCG CTT TCA TGT G

PGK1-R: GGCTCGGCTTTAACCTTGTTCC

IEX1-F: AGC CGC AGG GTT CTC TA

IEX1-R: GAT GGT GAG CAG CAG AAA GA.

PowerUp SYBR Green Master Mix (Thermo Fisher Scientific #A25742) was used for the detection of Real-Time PCR amplification on the Quant Studio 6 Flex Real-Time PCR system (Thermo Fisher Scientific #4485699).

## Supplementary Material

Supplementary information
