# Supplementary material for: Design of a 3D printed, motorized, uniaxial cell stretcher for microscopic and biochemical analysis of mechanotransduction
Source: Biol Open. 2021 Feb 10;10(2):bio057778. doi: 10.1242/bio.057778 (PMC7888744; doi:10.1242/bio.057778)
Supplement: Supplementary information [file biolopen-10-057778-s1.pdf]

Biology Open (2021): doi:10.1242/bio.057778 Supplementary information

Supplementary Figure 1

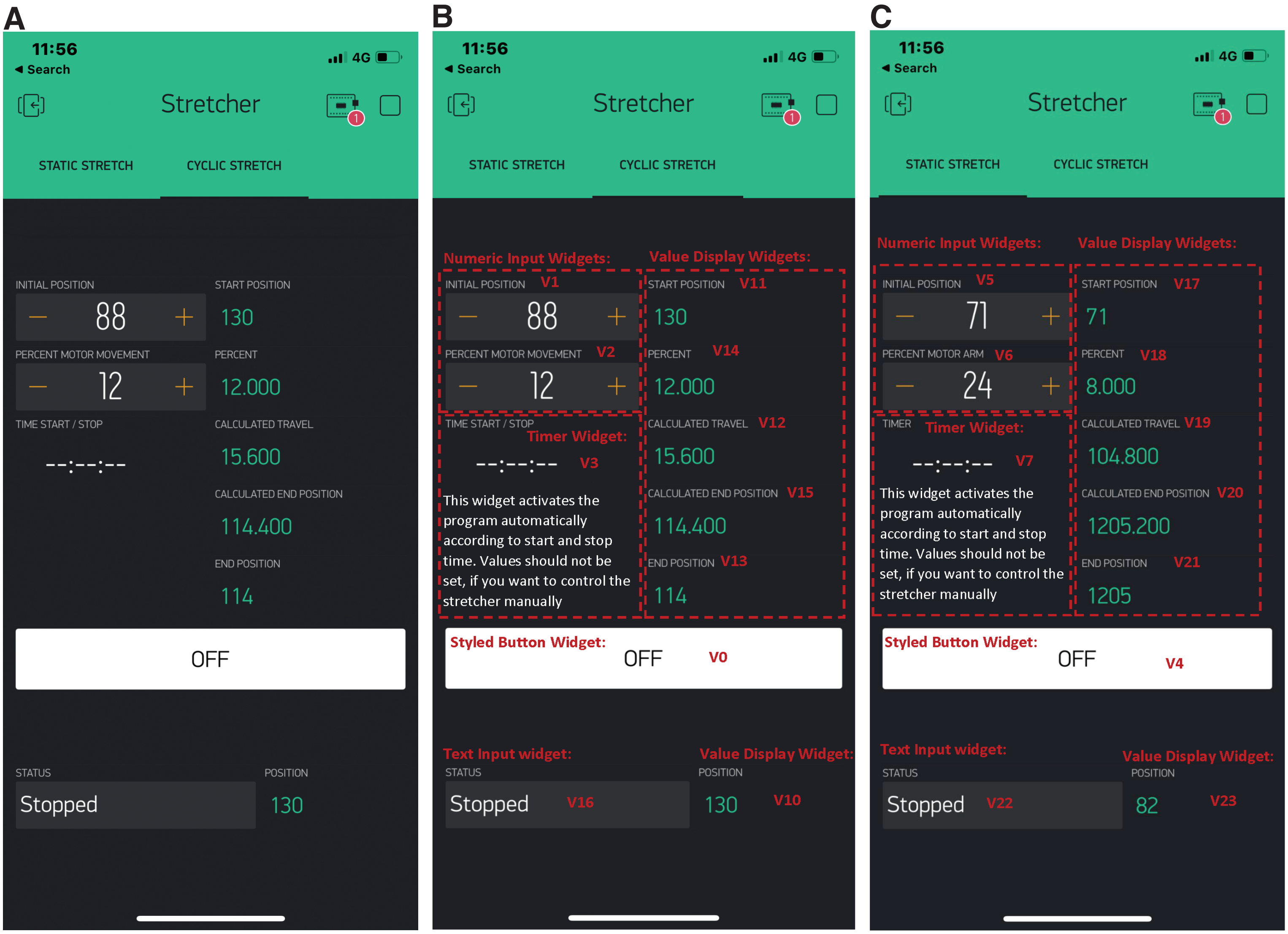

Figure S1. WiFi control of the stretcher.

The Blynk application was used to setup a control interface for the WiFi operation of the stretcher. The application can, in principle, be run on Android or iOS. We have successfully tested the app on iOS, both iPhone and iPad. (A) The user interface was designed to allow for input of stretch parameters. The “Initial Position” tab is used to adjust the membrane to zero stretch after it has been mounted, in case there is slack. The “Percent motor movement” indicated the amount of stretch. In our case, each percent reflects 0.5 mm movement. (B) Highlighted in red are the types of widgets used and the virtual pin (V) appointed to each. These correspond to the different inputs the hardware (ESP8266) receives from the ***cyclic*** stretching application, via the Blynk server. (C) Highlighted in red are the types of widgets used and the virtual pin (V) appointed to each. These correspond to the different inputs the hardware (ESP8266) receives from the ***static*** stretching application, via the Blynk server.

Biology Open • Supplementary information

Supplementary Figure 2

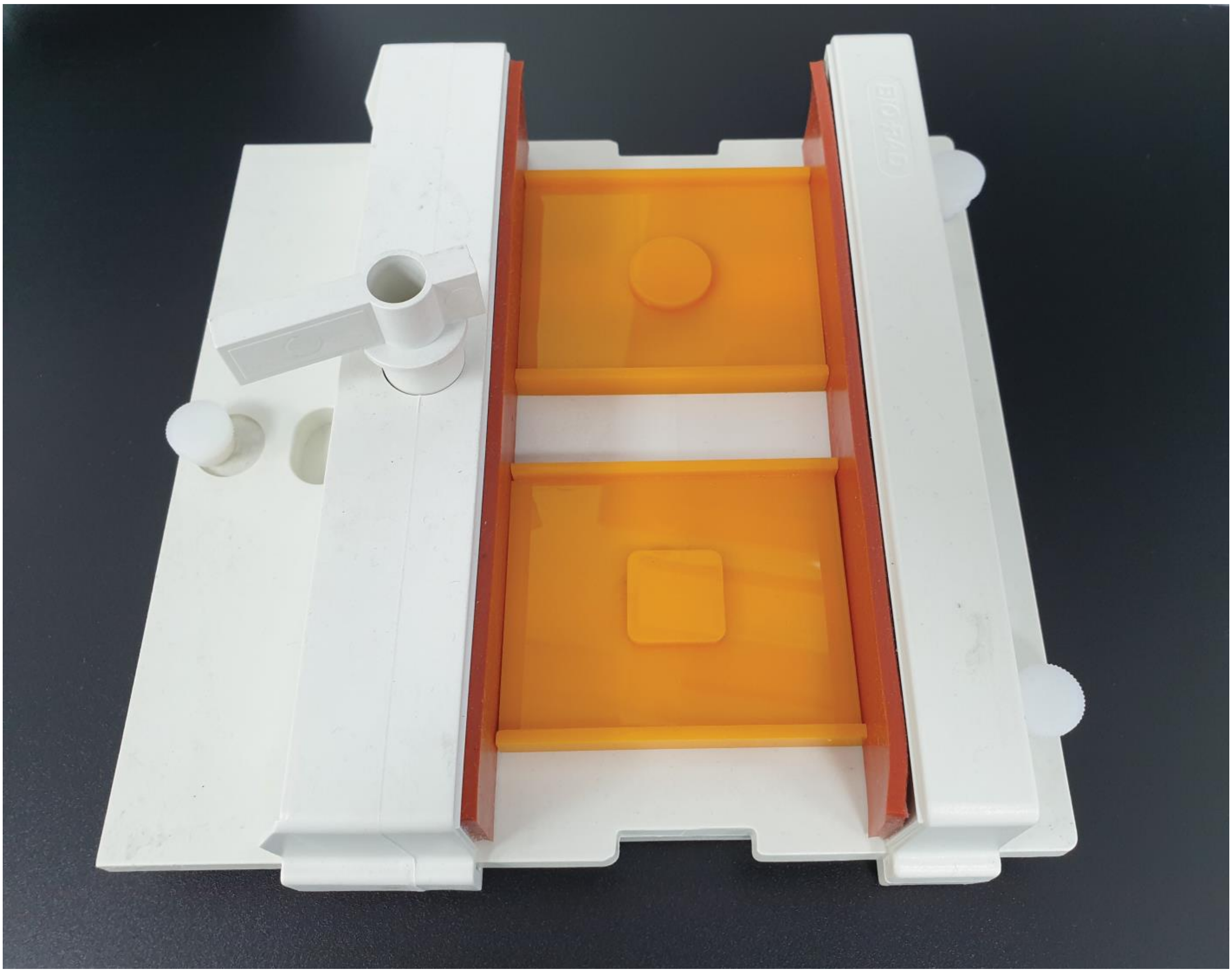

Figure S2. PDMS casting setup

PDMS casting molds are clamped into a BioRad mini agarose gel clamping system to pour the PDMS membranes which are left in the clamping system until cured.

Supplementary Figure 3

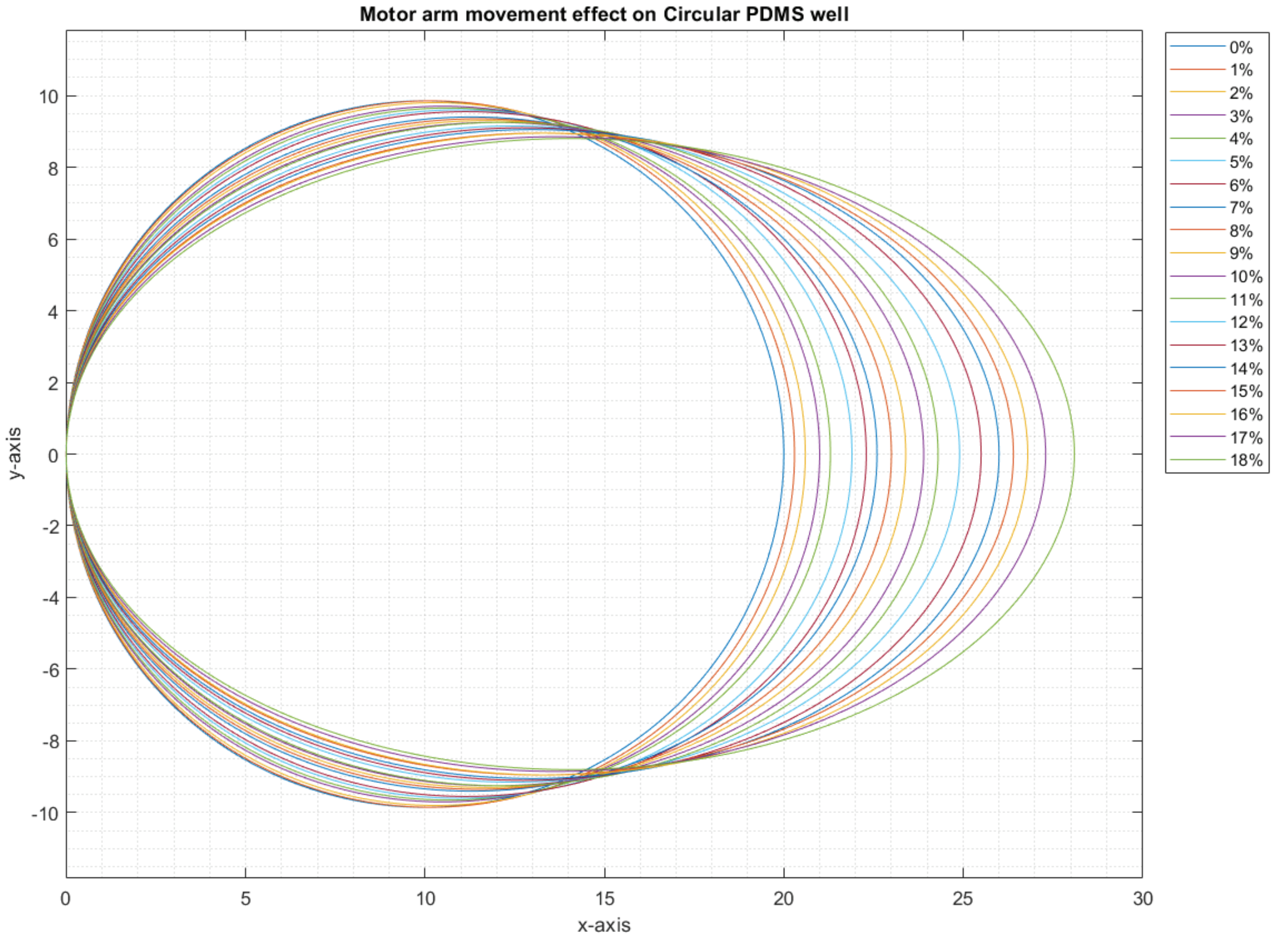

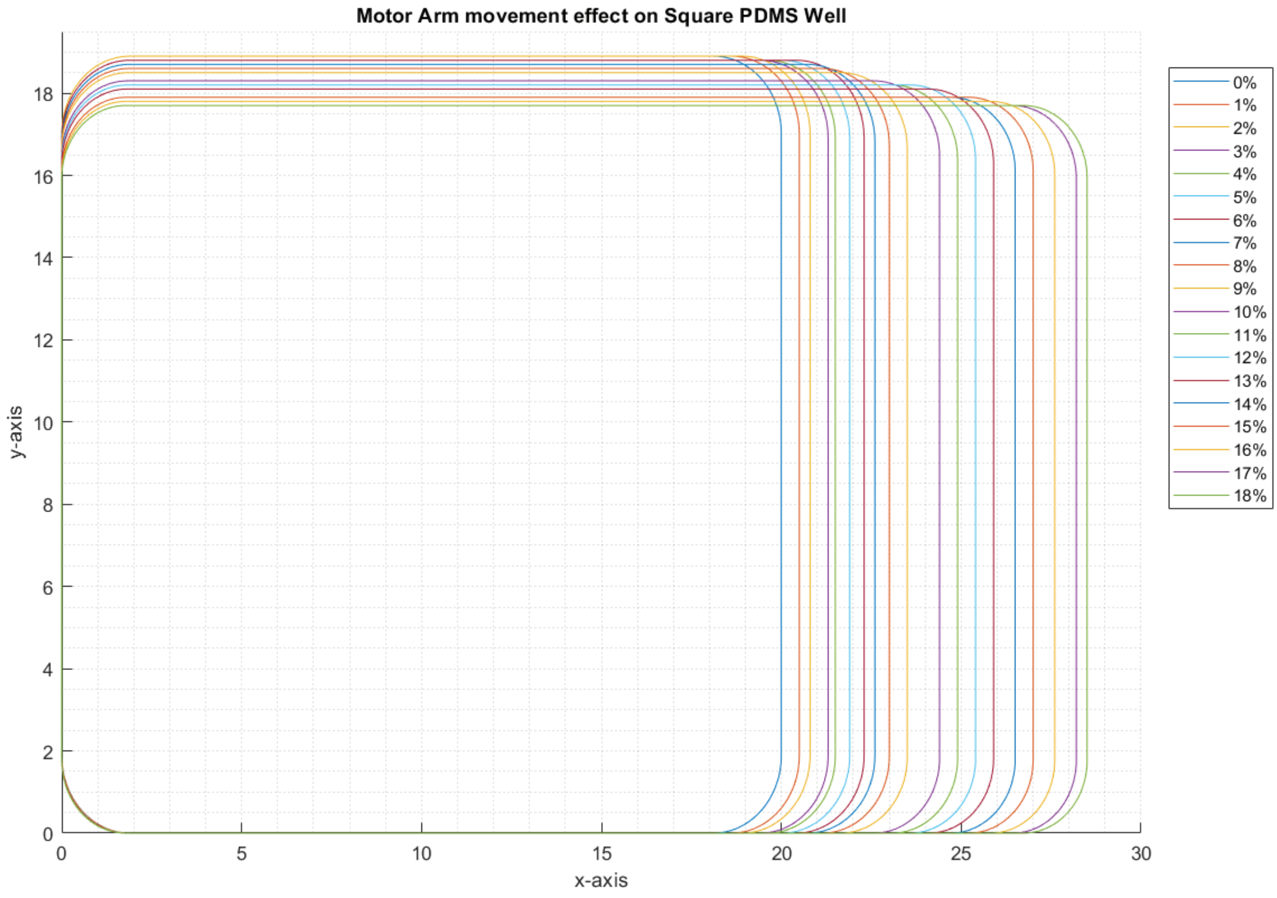

Biology Open • Supplementary information

Figure S3. Stretching parameters of round and square membranes.

Graph of the membrane outlines for circular (top) and square (bottom) membranes over the incremental stretching protocol. Every percent stretch represents 0.5 mm stretch.

Biology Open • Supplementary information

Supplementary Figure 4

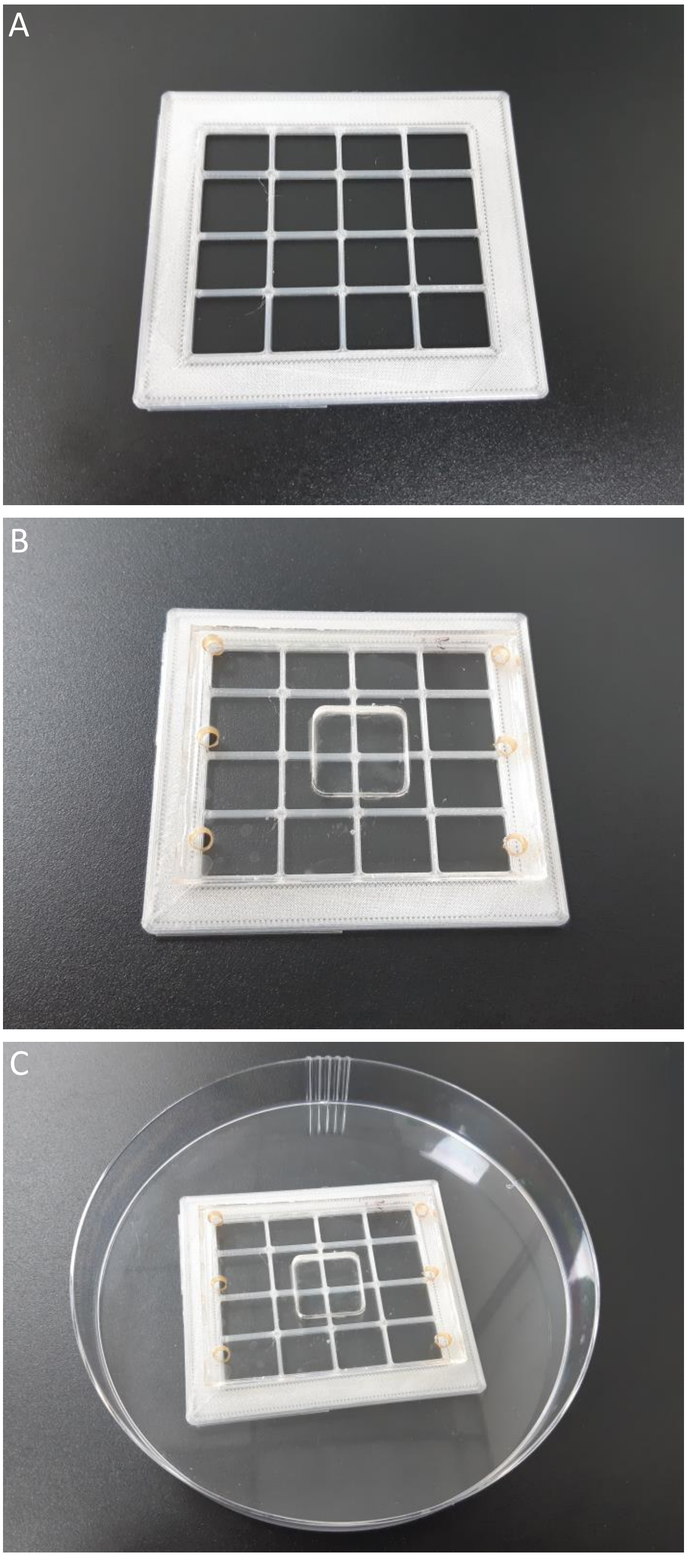

Figure S4. PDMS membrane support.

(A) A support grid was 3D printed to support the PDMS membrane while not attached to the stretcher. This support kept the well flat to ensure homogeneous cell plating and also allowed for the easy manipulation of the PDMS membrane when not attached to the stretcher. (B) The same support as in (A) pictured with a PDMS membrane resting on top. (C) The support and PDMS membrane in a 150 mm cell culture plate. 150 mm plates are used to enclose the membrane when coating with fibronectin, and also when culturing cells.

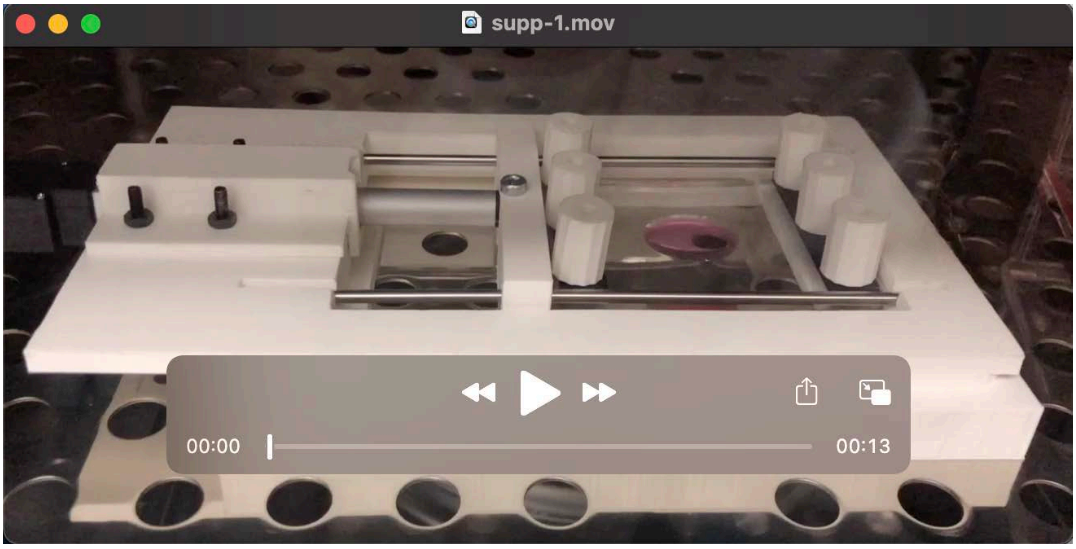

Movie 1

This movie shows the stretcher performing cyclic stretch.

Biology Open • Supplementary information

Table S1. Cost breakdown of the stretcher by components.

Part	Weight of material (g)	Cost (USD)
Stretcher insert	60 g	3.99
Motor hand	11 g	0.73
Motor Casing	9 g	0.59
PDMS washer	4 x 2 g	0.53
Nut Fastener	1 x 6 g	0.39
Stretcher incubator support	61 g	4.06
Membrane support	10 x 3 g	1.99
Ardunino Case	Top cover 4 g<br>bottom cover 6 g	0.66
Arduino	7	10.99
Acturonix Motor	40	70
PDMS mold	17 g	1.13
Wires	3 wires	1.34
Nuts & bolts	10 pieces each	0.73
Stainless Steel 3mm rods	2	1.25
Total Cost		98.44
